# Sodium overload due to a persistent current that attenuates the arrhythmogenic potential of a novel LQT3 mutation

**DOI:** 10.3389/fphar.2013.00126

**Published:** 2013-10-01

**Authors:** Adrien Moreau, Andrew D. Krahn, Pascal Gosselin-Badaroudine, George J. Klein, Georges Christé, Yohann Vincent, Mohamed Boutjdir, Mohamed Chahine

**Affiliations:** ^1^Centre de Recherche de l'Institut Universitaire en Santé Mentale de Québec, Quebec CityQC, Canada; ^2^Division of Cardiology, University of British Columbia, VancouverBC, Canada; ^3^Division of Cardiology, University of Western Ontario, LondonON, Canada; ^4^Laboratoire de Neurocardiologie, Université LyonLyon, France; ^5^VA New York Harbor Healthcare System, SUNY Downstate Medical Center and NYU School of Medicine, New York CityNY, USA

**Keywords:** cardiac arrhythmia, electrophysiology, Na_v_1.5, long QT syndrome, LQT3, sodium overload, heart

## Abstract

Long QT syndrome (LQTS) is a congenital abnormality of cardiac repolarization that manifests as a prolonged QT interval on 12-lead electrocardiograms (ECGs). The syndrome may lead to syncope and sudden death from ventricular tachyarrhythmias known as *torsades de pointes*. An increased persistent Na^+^ current is known to cause a Ca^2+^ overload in case of ischemia for example. Such increased Na^+^ persistent current is also usually associated to the LQT3 syndrome. The purpose of this study was to investigate the pathological consequences of a novel mutation in a family affected by LQTS. The impact of biophysical defects on cellular homeostasis are also investigated. Genomic DNA was extracted from blood samples, and a combination of PCR and DNA sequencing of several LQTS-linked genes was used to identify mutations. The mutation was reproduced *in vitro* and was characterized using the patch clamp technique and *in silico* quantitative analysis. A novel mutation (Q1476R) was identified on the *SCN5A* gene encoding the cardiac Na^+^ channel. Cells expressing the Q1476R mutation exhibited biophysical alterations, including a shift of SS inactivation and a significant increase in the persistent Na^+^ current. The *in silico* analysis confirmed the arrhythmogenic character of the Q1476R mutation. It further revealed that the increase in persistent Na^+^ current causes a frequency-dependent Na^+^ overload in cardiomyocytes co-expressing WT and mutant Na_v_1.5 channels that, in turn, exerts a moderating effect on the lengthening of the action potential (AP) duration caused by the mutation. The Q1476R mutation in *SCN5A* results in a three-fold increase in the window current and a persistent inward Na^+^ current. These biophysical defects may expose the carrier of the mutation to arrhythmias that occur preferentially in the patient at rest or during tachycardia. However, the Na^+^ overload counterbalances the gain-of-function of the mutation and is beneficial in that it prevents severe arrhythmias at intermediate heart rates.

## Introduction

In cardiomyocytes, action potentials (AP) ensure the propagation of excitation and the triggering of contraction. AP characteristics are directly related to the activity of voltage-gated sodium (Na^+^), calcium (Ca^2+^), and potassium (K^+^) channels (Amin et al., 2010). After a slight depolarization, voltage-gated Na^+^ channels (Na_v_1.5), encoded by the *SCN5A* gene, activate, which leads to an inward Na^+^ flow followed by a fast depolarization (Gellens et al., 1992). The AP is composed of a maintained depolarization, the “plateau” phase, followed by a final repolarization. The depolarization at the plateau is supported by a slowly decaying inward calcium current, background inward currents, and a persistent sodium current. The plateau is also supported by a delay in the onset of repolarizing potassium currents. The late activation of these potassium currents ensures final repolarization and causes the membrane potential to return to the diastolic level after about 400 ms. The maintenance of action potential duration (APD) is critically related to the balance between depolarizing currents and repolarizing currents, so that an increase in depolarizing current(s) or a decrease in repolarizing current(s) will increase APD and, consequently, the QT interval of the electrocardiogram (ECG).

Long QT syndrome (LQTS) includes a congenital abnormality of cardiac repolarization that causes syncope and sudden death (Huang et al., 2011). This hereditary cardiac disease manifests as an increase in the QTc value over 450 ms on 12-lead ECGs, which points to an increase in the duration of ventricular APs. Under optimal conditions, QTc prolongation can induce ventricular tachyarrhythmia called *torsade de pointes* (TdP), which can degenerate into lethal ventricular fibrillation. To date, mutations in thirteen genes have been identified as causing 13 types of LQTS (LQT1-13) (Schwartz et al., 2012) and are divided into three major groups: (1) mutations in genes encoding voltage-gated channels [*KCNQ1* (LQT1), *KCNH2* (LQT2), *SCN5A* (LQT3), *KCNJ2* (LQT7), *KCNJ5* (LQT13)], (2) mutations in genes encoding subunits regulating voltage-gated channel [*KCNE1* (LQT5), *KCNE2* (LQT6), *SCN4B* (LQT 10)], and (3) mutations in genes encoding channel interacting proteins [*ANKB* (LQT4), *KCNE1* (LQT5), *KCNE2* (LQT6), *CAV3* (LQT9)].

*SCN5A* gene mutations can lead to several inherited cardiac diseases such as LQT3, Brugada syndrome (BrS) (Moreau et al., 2012), progressive cardiac conduction defect (CCD), sinus node disease, and dilated cardiomyopathy (DCM) (Amin et al., 2010; Gosselin-Badaroudine et al., 2012). In LQT3, a gain-of-function mediated by the appearance of a persistent current usually characterizes Na^+^ channel mutations. The persistent Na^+^ current has previously been shown to induce Ca^2+^ overload (Tang et al., 2012). Indeed, in hypoxia condition, the persistent Na^+^ current is increased and have been identified as a predominant cause of the deleterous Ca^2+^ overload (Tang et al., 2012). Such phenomenon could be important in the development of LQT3 syndrome and would be driven by the activation of Na^+^/H^+^ and Na^+^/Ca^2+^ exchangers. These exchangers are predominantly implicated in the Ca^2+^, Na^+^, and pH regulation. However, most mutations in K^+^ channels or their regulatory subunits cause a loss-of-function characterized by a dominant negative effect. These biophysical defects lead to AP prolongation, creating a substrate for the development of ventricular arrhythmias (Schwartz et al., 2012).

The purpose of our study was to investigate the impact of a mutation causing LQTS in a patient and her family members using an *in vitro* heterologous expression system, the voltage-clamp technique, and *in silico* analysis. The biophysical investigation of the mutation in tsA201 cells revealed a positive shift in steady-state (SS) inactivation that results in an increased window current associated with the presence of a persistent Na^+^ current, which would explain the link between the mutation and LQTS in the family members. Furthermore, *in silico* experiments revealed that the persistent Na^+^ current induces an increase in the intracellular Na^+^ concentration. This causes an increase in repolarizing currents that shortens APD, counterbalancing the direct effect of the gain-of-function caused by the mutation and potentially benefitting the patient.

## Methods

### Clinical evaluation

The proband and her family members underwent a detailed clinical and cardiovascular examination, including a 12-lead ECG, a determination of hemodynamic parameters, and laboratory blood tests. Echocardiography was used to exclude any structural heart disease. The QT interval was measured in lead II or V5 of the ECG and was corrected for the heart rate (QTc) using Bazett's formula. Exercise testing was performed to detect QT adaptation to heart rate changes, which is known to predict the genotype and direct therapy in LQTS (Schwartz et al., 2001; Wong et al., 2010).

The investigation conformed to the principles set out in the Declaration of Helsinki and was approved by the local institutional ethics committees on human subject research. The participants provided written informed consent prior to the study.

### Genotyping

The samples were sent to Genaissance Familion® (New Haven, CT, USA) for genetic testing of inherited cardiac syndromes. Genomic DNA was extracted and was amplified by PCR. Templates of the target exons, splice junctions, and flanking regions of *KCNQ1* (LQT1), *KCNH2* (LQT2), *SCN5A* (LQT3), *KCNE1* (LQT5), *KCNE2* (LQT6), *CACNA1C* (LQT8, exons 8 and 9), *CAV3* (LQT 9), *SCN4B* (LQT 10), *AKAP9* (LQT 11, exon 18), and *SNT12* (LQT 12) were generated and genotyped.

### Mutagenesis

Mutant Na_v_1.5/Q1476R was generated using QuickChange TM site-directed mutagenesis kits according to the manufacturer's instructions (Stratagene, La Jolla, CA, USA). The hNa_v_1.5/mutants were constructed from WT channels (NCBI accession number: M77235) using the following mutagenic sense and antisense primers (mutated sites are underlined):

1. 5′-CAACTTCAACCAACGGAAGAAAAAGTTAGGG-3′
2. 5′-CCCTAACTTTTTCTTCCGTTGGTTGAAGTTG-3′

Mutant and the wild-type (WT) Na_v_1.5 in a pcDNA3 construct were purified using Qiagen columns (Qiagen Inc., Chatsworth, CA, USA).

### Transfection of the tsA201 cell line

TsA201 is a mammalian cell line derived from human embryonic kidney HEK 293 cells by stable transfection with SV40 large T antigen (Margolskee et al., 1993). The tsA201 cells were grown in high glucose DMEM supplemented with fetal bovine serum (10%), L-glutamine (2 mmol/L), penicillin (100 U/ml), and streptomycin (10 mg/ml) (Gibco BRL Life Technologies, Burlington, ON, Canada) and were incubated in a 5% CO_2_ humidified atmosphere. The cells were transfected using the calcium phosphate method (Margolskee et al., 1993), with the following modification to facilitate the identification of individual transfected cells: the cells were co-transfected with an expression plasmid for a lymphocyte surface antigen (CD8-a) (Jurman et al., 1994). The human Na^+^ channel β_1_ subunit and CD8 were constructed in the piRES vector (piRES/CD8/β_1_). With this strategy, transfected cells that bind beads also express the β_1_-subunit. cDNA (5 μ g) coding for the WT or mutant Na^+^ channel α-subunit and 5 μ g of piRES/CD8/β_1_ were used. For the patch clamp experiments, 2 to 3-day-post-transfection cells were incubated for 5 min in a medium containing anti-CD8-a coated beads (Jurman et al., 1994) (Dynabeads M-450 CD8-a). Unattached beads were removed by washing. The beads were prepared according to the manufacturer's instructions (Dynal A.S., Oslo, Norway). Cells expressing surface CD8-a fixed the beads and were visually distinguishable from non-transfected cells by light microscopy.

### Patch clamp technique

Macroscopic Na^+^ currents from tsA201-transfected cells were recorded using the whole-cell configuration of the patch clamp technique (Hamill et al., 1981). Patch electrodes were made from 8161 Corning borosilicate glass and were coated with HIPEC (Dow-Corning, Midland, MI, USA) to minimize their capacitance. Patch-clamp recordings were made using low resistance electrodes (<1 MΩ), and a routine series resistance compensation by an Axopatch 200 amplifier (Axon Instruments, Foster City, CA, USA) was performed to values >80% to minimize voltage-clamp errors. Voltage-clamp command pulses were generated by microcomputer using pCLAMP software v10.0 (Axon Instruments). Na^+^ currents were filtered at 5 kHz, digitized at 10 kHz, and stored on a microcomputer equipped with an AD converter (Digidata 1300, Axon Instruments).

### Solutions and reagents

For whole cell recordings, the patch pipette contained (in mmol/L): 35 NaCl, 105 CsF, 10 EGTA, and 10 Cs-HEPES. The pH was adjusted to 7.4 using 1 N CsOH. The bath solution contained (in mmol/L) 150 NaCl, 2 KCl, 1.5 CaCl_2_, 1 MgCl_2_, 10 glucose, and 10 Na-HEPES. The pH was adjusted to 7.4 with 1 N NaOH. The recordings were made 5 min after establishing the whole cell configuration to allow the current to stabilize and to permit adequate diffusion of the contents of the patch electrode. All the recordings were made in the following order: I/V curve, SS inactivation, and recovery from inactivation. Experiments were carried out at room temperature (22–23°C).

#### In silico analysis and AP modeling

The Priebe and Beuckelmann (1998) (PB), the Iyer et al. (2004) (IMW) model and the Ten Tusscher et al. (2004) (TNNP) models were considered as possible alternatives to represent human ventricular myocyte (Priebe and Beuckelmann, 1998; Iyer et al., 2004; Ten Tusscher et al., 2004). A comparison of these models was done by Ten Tusscher et al. in 2006 and it showed that the TNNP model was the most pertinent to reproduce the effects of IKr and IKs block on the human ventricular AP duration (Ten Tusscher et al., 2006). We considered it as a valid model to address changes related to LQT conditions. Besides, rather than using Markov models, this model is based on Hodgkin–Huxley equations, which are easy to adjust to known experimental data (Fink and Noble, 2009).

Thus, the *in silico* analysis was performed with the Ten Tusscher et al. (2004) model (Ten Tusscher et al., 2004), using the tentusscher_noble_noble_panfilov_2004_a.cellml file, ran in the COR environment (Peter Garny, http://cor.physiol.ox.ac.uk/).

Changes from the original set of equations are described in the Appendix.

When we attempted to modify the activation and inactivation parameters of the Na^+^ current (INa) in the original TNNP model, instabilities were found, which could not be resolved. Thus, the mathematical equations used for activation, inactivation, and recovery from inactivation of INa were taken from a stable guinea-pig ventricular cell model adjusted for 37°C (Pasek et al., 2008). However, the processes were accelerated two-fold (the time constants were reduced two-fold). This was done to allow the model to reproduce the experimentally observed Na^+^ currents when we ran, in the model set for room temperature, the same voltage-clamp protocols as used in our *in vitro* experiments.

To adapt to the formalism of the Na^+^ current (INa) calculation that uses m^3^, the measured values of the SS apparent activation variable measured in tsA201 cells were fitted with the following equation: *m*_inf_ = [1/(1 + exp((*V*_m_ − *V*_h_)/*k*))]^3^, which yielded values of *V*_h_ = −59.1 mV and *k* = −7.9 mV for WT and *V*_h_ = −56.8 mV and *k* = −8.7 mV for Q1476R. This explains why these values of *V*_h_ and *k* were different from those calculated by fitting a simple Boltzmann function to data measured in tsA201 cells (Table 1).

**Table 1:** **Biophysical properties of Na_v_1.5/WT and Q14767R**.

		**WT (*n* = 13)**	**Q1476R (*n* = 14)**
Peak current (pA/pF)		−739.1 ± 73.1	−690.8 ± 69.9
Persistent current (% I Na^+^)		0.36 ± 0.08	0.74 ± 0.11^*^
Activation	*V*_1/2_ (mV)	−47.9 ± 1.2	−46.3 ± 1.1
	*k* (mV)	−5.6 ± 1.2	−6.5 ± 0.4
Inactivation	*V*_1/2_ (mV)	−92.0 ± 1.3	−85.5 ± 0.4^*^
	*k* (mV)	4.4 ± 0.1	4.3 ± 0.1
Recovery from fast inactivation	*A*	99.9 ± 0.05	98.7 ± 1.1
	Tau	2.7 ± 0.1	2.2 ± 0.3

V_1/2_, Mid-point for activation or inactivation.

k, Slope factor for activation or inactivation.

* Difference between Na_v_1.5/WT and Na_v_1.5/Q1476R (p < 0.05).

Lastly, 5.5 mV was added to *V*_h_ and 0.5 mV to k to account for the difference in temperature given that the experiment was conducted at room temperature whereas the model was designed to work at 33°C, such that *V*_h_ = −59.1 + 5.5 and *k* = −7.9 + 0.5 for WT and *V*_h_ = −56.8 + 5.5 and *k* = −8.7 + 0.5 for Q1476R.

The SS inactivation functions were *h*_inf_ = 1/(1 + exp((*V*_m_ − *V*_h_)/*k*)), where *V*_h_ = −92.0 + 5.5 and *k* = 4.4 for WT, and *V*_h_ = −86.1 + 5.5 and *k* = 4.3 for Q1476R. In addition, 5.5 mV was added to *V*_h_ to account for the difference in temperature.

This adjustment of the kinetic parameters was derived from the detailed analysis of the effects of temperature on the activation and inactivation kinetics of Na_v_1.5 channels by Nagatomo et al. (1998). It reflects a change in temperature from 23 to 33°C. A linear extrapolation to 37°C could have been done. Nevertheless, no differences should be observed as these corrections would apply to both WT and mutated fast INa model equations.

The modeling of the heterozygous condition is based on the assumption that, in the carrier, both the WT and the mutated genes would lead to the presence of equal amounts of each corresponding channel protein. Thus, half of the fast Na^+^ current is characterized by the parameters of the WT channels while the second half is characterized by the parameters of the Q1476R channels.

The persistent part of the fast Na^+^ current was initially absent in the TNNP model. A persistent current was added that would not inactivate but that would activate and deactivate following the same equations as the fast Na^+^ current (see equations in the Appendix).

Initially, the model was run with the basic set of values of variables and constants as used in the TNNP 2004a model, except for the formulation of INa, as described above. The APD_90_ was measured as the time between upstroke and 90% repolarization from the highest level of the plateau. No attempt was made to readjust the APD_90_ at SS at 1 Hz, which was increased from 322 ms in the original TNNP model to 454 ms for our WT settings.

Voltage-clamp testing was performed using MATLAB to show that the basic properties of the fast INa current, the persistent Na^+^ current and the window current, adjusted for room temperature, matched those of the WT and mutated currents as determined in the expression experiments when using the same protocols and measurement procedures in the presence of a realistic series resistance (not shown).

Based on our previous modeling experience with a persistent INa (Christe et al., 2008), it was necessary to allow the model to stabilize with a small amount of persistent INa before increasing the persistent INa until reaching at most one-third of the fraction of the fast peak INa observed in expression experiments. This was the maximal amount of persistent INa tolerated by the heterozygous mutation model in order to ensure that a stable AP was generated at 1 and 1.5 Hz.

### Data analysis and statistics

The electrophysiological data were analyzed using macros in Clampfit (pCLAMP v10.0, Molecular Devices) and custom programs written using MATLAB (The MathWorks Inc.). Data are expressed as means ± sem (standard error of the mean). Statistical comparison were performed using an unpaired Student's *t*-test or One-Way Anova with Bonferroni *post-hoc* test in Sigma plot (Jandel Scientific software). Differences were deemed significant at a *P*-value < 0.05.

## Results

### Clinical characteristics of the index patient and the family members

**Figure 1:**
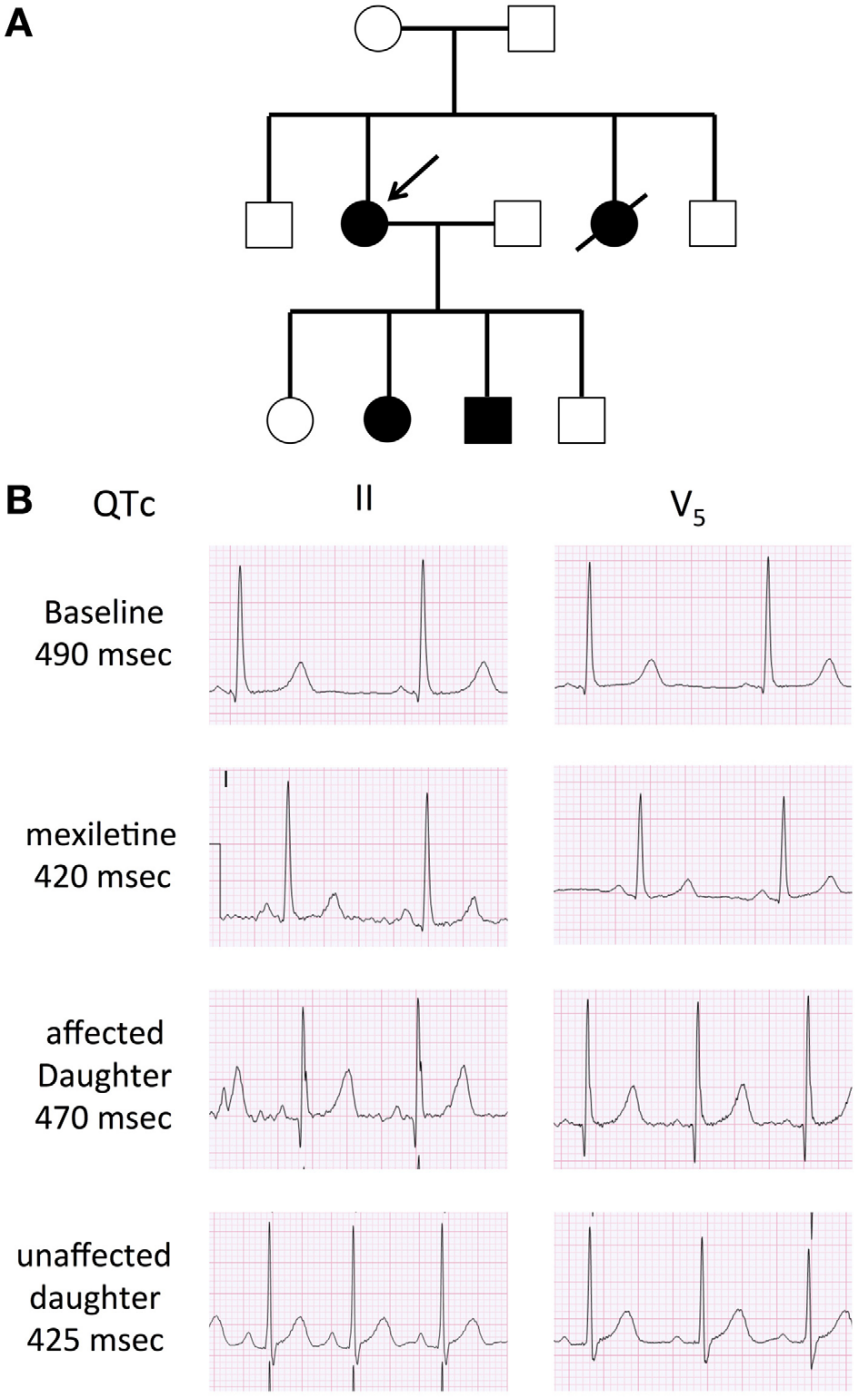
**Family pedigree and 12-lead ECG of the proband. (A)** Family pedigree. Subjects II-2 and III-2 and III-3 with the LQT3 phenotype (black circle and square), the arrow indicates the proband. **(B)** Twelve-lead ECG. Baseline ECG in the proband, demonstrating a long isoelectric ST segment, followed by a deferred T was with normal morphology. The second ECG represents a repeat ECG after 2 doses of 200 mg of mexilitine. The ECGs of her affected and unaffected daughter are also presented. Voltage scale: 0.5 mV/large square, time scale: 0.2 s/large square.

**Figure 2:**
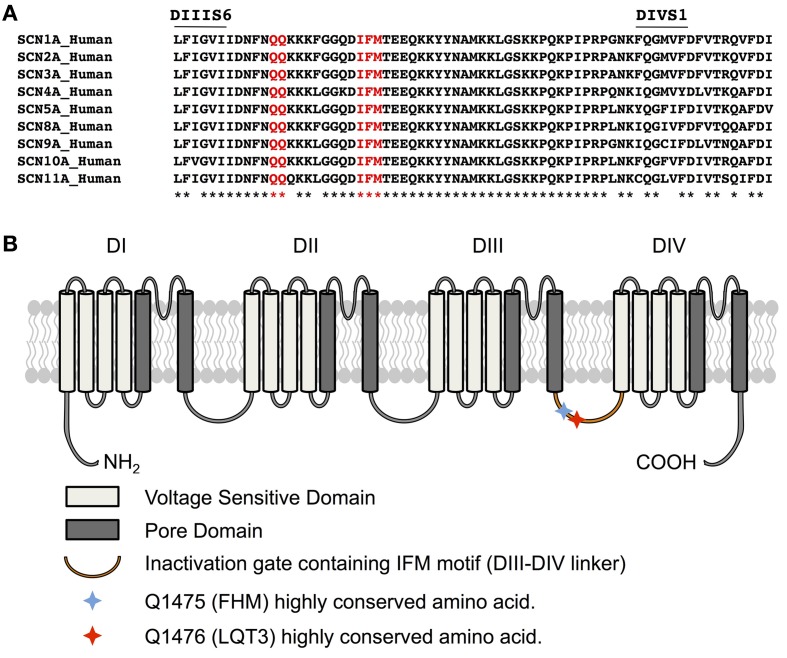
**Q1476 is within a highly conserved region. (A)** Amino acid alignments of the region between transmembrane domains IIIS6 and IVS1 (III-IV linker) showing in red that glutamine 1475 and glutamine 1476 are highly conserved in human voltage-gated sodium channels. The IFM inactivation particle is displayed in red. **(B)** Schematic representation of the Na_v_1.5 channel structure displaying voltage sensitive domains, the segments forming the pore domain, and the inactivation gate. Q1475 and Q1476 are also indicated. The missense mutation of one these two amino acids leads to several pathologies, depending on the channel impacted. The mutation of Q1475 in Na_v_1.1 causes familial hemiplegic migraine (FHM), whereas the mutation of Q1476 in Na_v_1.5 causes LQT3 syndrome.

A 34-year-old female was assessed, in the context of a family history of sudden death, for a remote history of syncope after awakening to answer the telephone. Her sister had experienced a series of similar episodes at age 14 and had subsequently collapsed at rest and could not be resuscitated. The autopsy did not reveal a cause of death. A maternal cousin and grandaunt also experienced sudden death (Figure 1A). The resting QTc of the proband ranged from 450 to 485 ms and shortened to 422 ms at peak exercise. The echocardiogram was normal. Nadolol 40 mg once daily and mexiletine 200 mg twice daily were initiated. While mexiletine shortened the QTc (Figure 1B), it was discontinued before reassessment because of side effects. We identified an A-to-G base change at nucleotide position 5300 in exon 25 on the *SCN5A* gene by PCR and DNA sequencing. The base change led to a glutamine (Q)-to-arginine (R) substitution at residue 1476. The Q1476 residue is in a region of the *SCN5A* gene/Na_v_1.5 protein in Na^+^ channels (Figure 2A) that has been extraordinarily highly conserved throughout evolution, from humans to zebra fish (not shown). Another highly conserved glutamine (Q1475, Figure 2A) was located upstream from the Q1476 residue. The mutation occurred in an important region of the Na^+^ channel known as the inactivation gate (Figure 2B). An H558R polymorphism was associated with the novel Q1476R mutation. The four children of the index patient were examined and presented QTc values of 414, 444, 480, and 502 ms. Three of them underwent an exercise test and their QTc values went from 444 to 442 ms, 480 to 500 ms, and 502 to 522 ms, respectively. The two who presented with borderline QTc prolongation were subsequently confirmed to be carrying the Q1476R variant. The index patient remained free of symptoms during the 5-year follow-up period.

### Biophysical characterization

**Figure 3:**
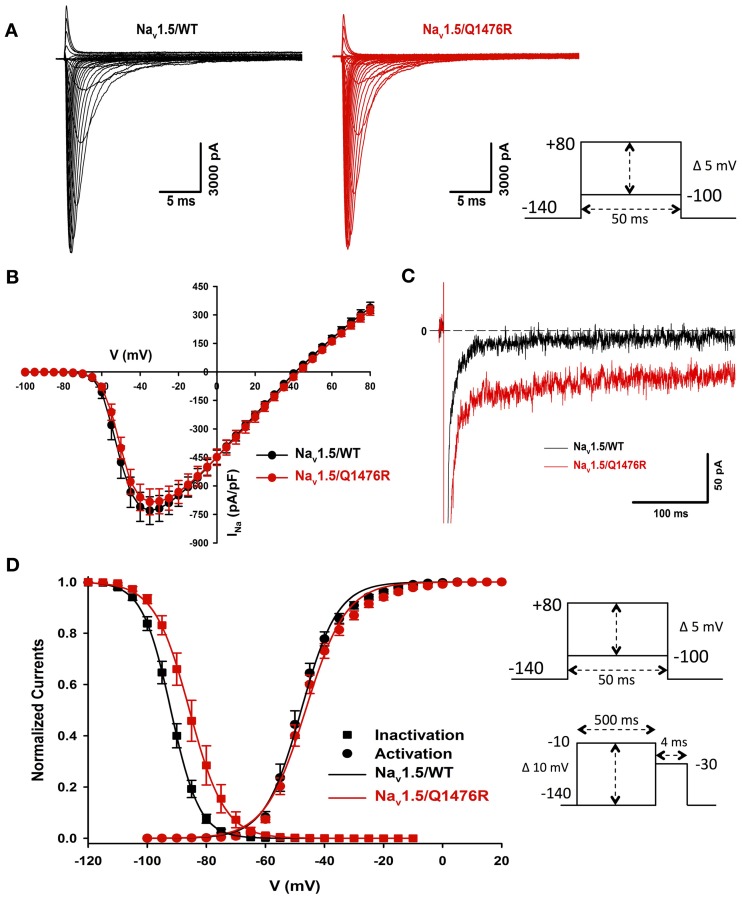
**Biophysical characterization of Na_v_1.5/WT and the Q1476R mutant channels. (A)** Representative whole-cell current traces of the WT (left) and Q1476R (right) channels. Currents were elicited using a voltage-clamp protocol where depolarizing pulses were applied for 50 ms from −100 to +80 mV in 5 mV increments (see protocol in inset). The zero current corresponds to the basal line shown at the beginning of each trace. **(B)** After normalization to cell capacitance, current-voltage (I-V) relationships were constructed for the Na_v_1.5/WT (*n* = 13, black) and Q1476R (*n* = 14, red) channels. **(C)** Representative current traces obtained after a −30 mV depolarizing pulse showing INap. The Na_v_1.5/WT channel trace is depicted in black while the Na_v_1.5/Q1476R channel trace is depicted in red. **(D)** Voltage dependence of SS activation (circles) or inactivation (squares) for the WT (black) and Q1476R (red) Na_v_1.5 Na_v_1.5 channels. A standard Boltzmann function was used to fit activation curves and to obtain the *V*_1/2_ and given in Table 1 [*G*(*V*)/*G*_max_ = 1/(1 + exp(−(*V* − *V*_1/2_)/*k*))]. Inactivation currents were obtained by applying conditioning pre-pulses to membrane potentials ranging from a holding potential of −140 to −10 mV for 500 ms in 5 mV increments and were then measured using a 4-ms pulse to −30 mV at each step (see protocol in inset). The recorded inactivation values were fitted to a standard Boltzmann equation [*I*(*V*)/*I*_max_ = 1/(1 + exp((*V* − *V*_1/2_)/*k*)) + *C*] to obtain the values given in Table 1.

**Figure 4:**
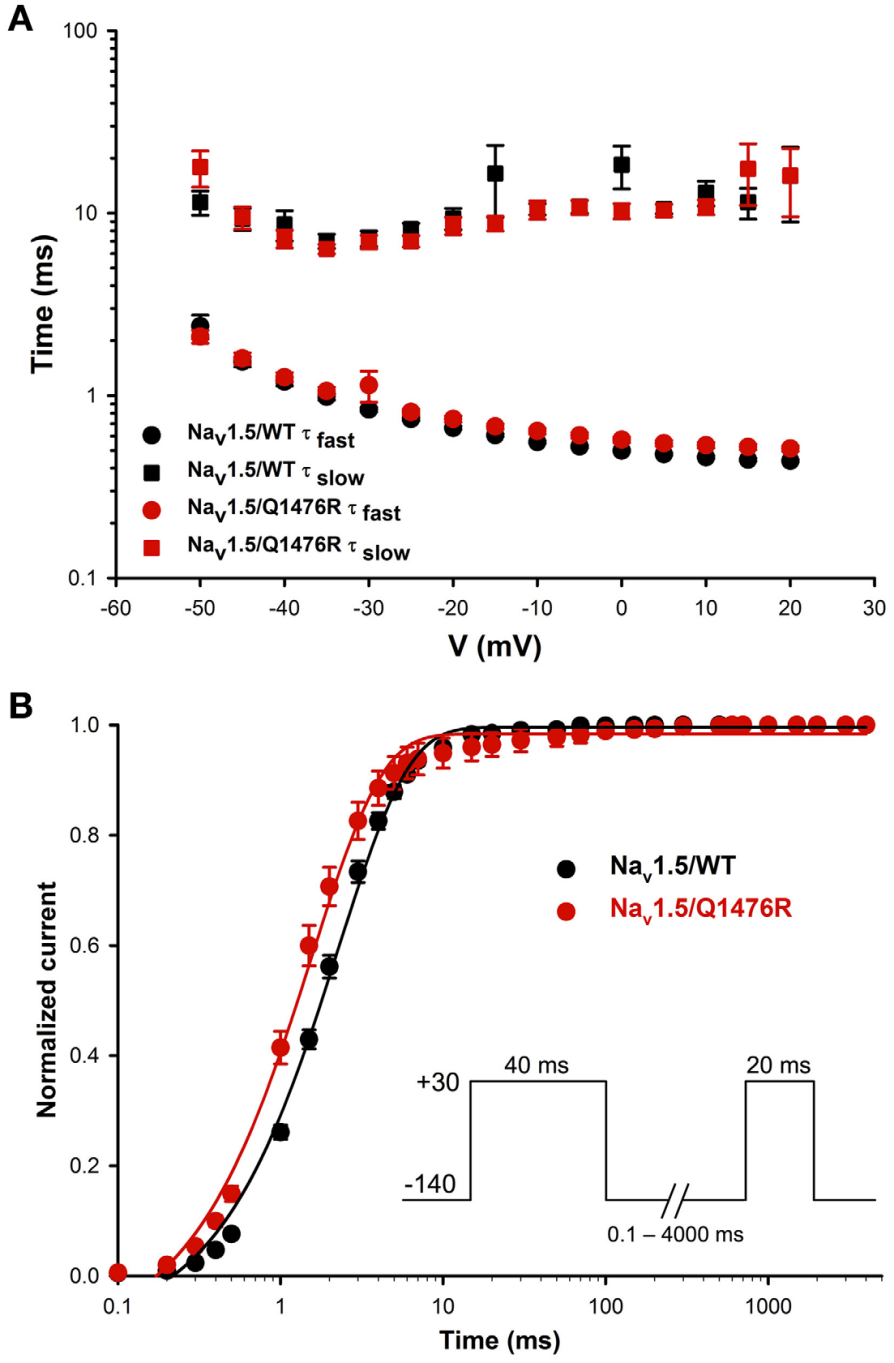
**Time constants of inactivation and of recovery from inactivation. (A)** Curves representing the time constant of inactivation as a function of the voltage for the WT and Q1476R channels. A two-exponential function was used to fit the inactivation curves observed on whole cell current traces: *I* = *A*_fast_(exp[−(*t* − *k*)/τ_fast_]) + *A*_slow_(exp[−(*t* − *k*)/τ_slow_] + *C*), where *A*_fast_ and *A*_slow_ are fractions of recovery of the fast and slow components, *t* is time, and *k* is the delay factor for activation or inactivation. τ_fast_ and τ_slow_ were obtained from the equation from −50 to +20 mV for the Na_v_1.5/WT and Q1476R channels. **(B)** Recovery from fast inactivation of the WT (black) and Q1476R (red) channels. Curves were obtained using the two-pulse protocol shown in the inset. The pulses (+30 mV) induced maximal activation, and the time interval between pulses represents the time needed to obtain a re-activation. The recorded recovery from inactivation values were fitted to a single exponential equation [A^*^(1 − exp(−*x*/τ) + *C*)] to obtain the values given in Table 1.

We investigated whether the novel mutation caused an alteration of the biophysical properties of the Na_v_1.5 Na^+^ channel that would explain the clinical phenotype of the index patient. Macroscopic Na^+^ currents were recorded from tsA201 cells expressing either WT (Na_v_1.5/WT) or mutant channels (Na_v_1.5/Q1476R) co-transfected with the regulatory β_1_-subunit (see Methods for more details) (Figure 3A). The mutant Na^+^ channel current exhibited no significant shift in the current-voltage (I/V) relationship, and the current density was similar to that of the WT channel (WT: −739.1 ± 73.1 pA/pF, Q1476R: −690.8 ± 69.9 pA/pF) (Figure 3B, Table 1). This result is based on the measurement of the peak current which is defined as the maximal absolute current obtained with the stimulation protocol (see Figure 3 legend). This peak current corresponds to the value where the global conductance reaches its maximum. A small persistent Na^+^ current was recorded. The persistent Na^+^ current was investigated using a 400 ms two-pulse protocol, and it was measured as mean of the current between 100 and 350 ms. The magnitude of this persistent current was significantly (*p* < 0.05) different from that recorded from WT channels at −30 mV (0.36 ± 0.08%, *n* = 13 for WT channels, 0.74 ± 0.11%, *n* = 14 for mutant channels) (Figure 3C, Table 1). SS activation (Gv) and inactivation curves were also studied (Figure 3D, Table 1). The Gv curve of the mutant Na^+^ channel showed no significant shift (*p* = 0.340 for *V*_1/2_ and *p* = 0.081 for the slope factor). However, there was a 6.5 mV (*p* < 0.05) shift in the SS inactivation curve toward more positive voltages for the Q1764R mutant channel (*V*_1/2_ Na_v_1.5/Q1476R = −85.5 ± 0.4 mV, *n* = 14 vs. *V*_1/2_ Na_v_1.5/WT = −92.0 ± 1.3 mV, *n* = 13). The slope factor was not significantly affected (*p* = 0.544). The mutation did not induce any significant changes in the time course of the Na^+^ current decay or the recovery from inactivation (Figure 4).

**Figure 5:**
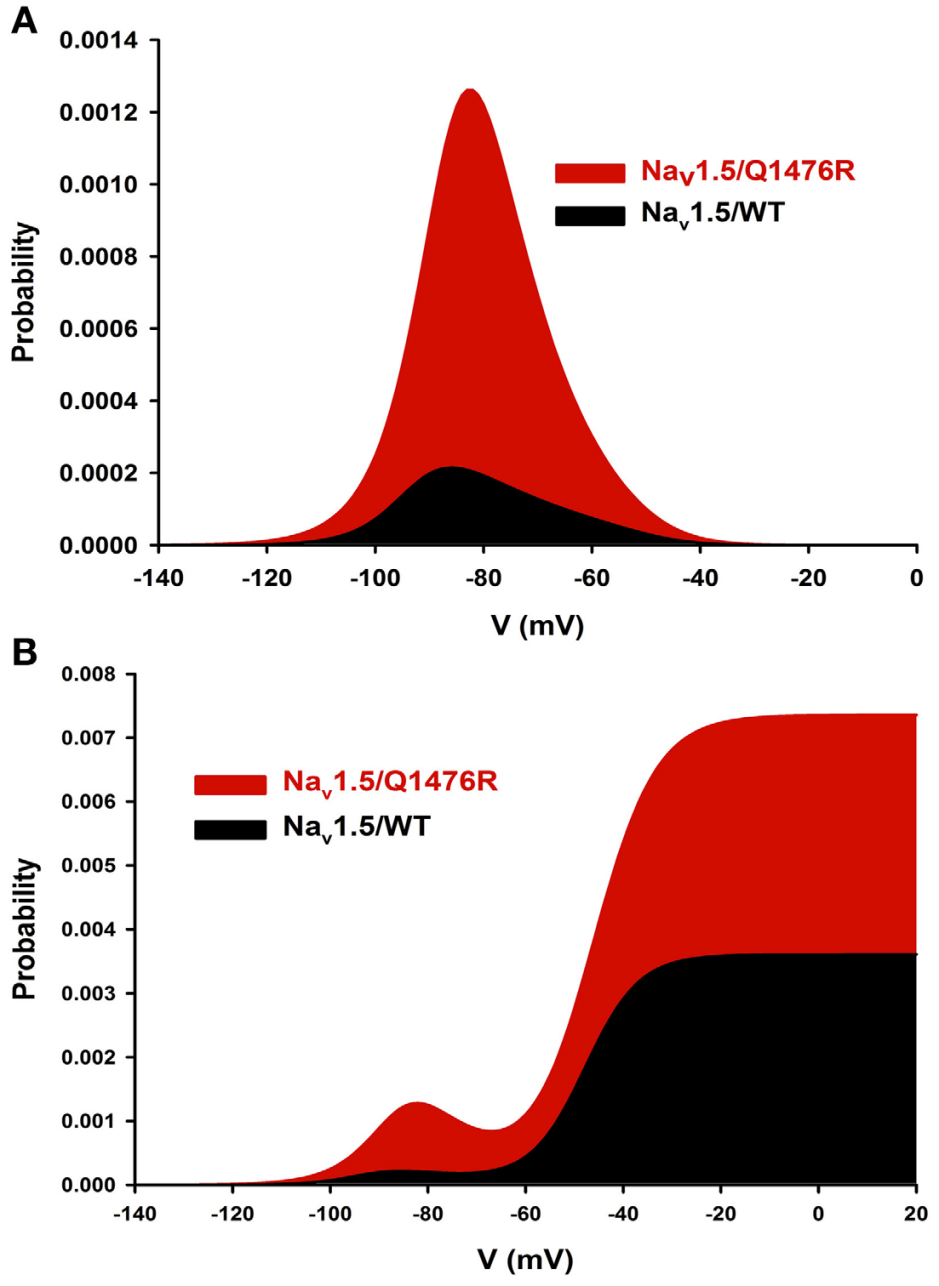
**Na_v_1.5/WT and Q1476R window currents. (A)** The window current is defined as the overlap between activation and inactivation. The window current was obtained using the following equation: (1/(1 + exp((*V*_1/2activation_ − *V*)/*k*_activation_)) × ((1 − *C*)/(1 + exp((*V* − *V*_1/2inactivation_)/*k*_inactivation_)) + *C*). **(B)** The INap component was added to the window current of the WT or the Q1476R channel.

The overlap of SS activation and inactivation of Na^+^ channels defines a range of voltages (i.e., window) where Na^+^ channels open, resulting in an inward Na^+^ current that could potentially depolarize the resting membrane potential and increase myocyte excitability. The Q1476R mutation induced an increase in the overlap of Na^+^ channel activation and inactivation by shifting the inactivation to more positive potentials. This shift tended to expand the window and thus, the fraction of permanently activated channels. Figure 5A shows the predicted window currents of the WT and Q1476R mutant Na_v_1.5 channels. The Q1476R mutation produced a ~7-fold increase in the Na_v_1.5 window current, suggesting that more channels had reopened within the window region. Figure 5B shows the increase in the window current together with the increase in the perisistent component, suggesting that Q1476R had a higher probability to open at voltages ranging from −140 to +20 mV.

**Figure 6:**
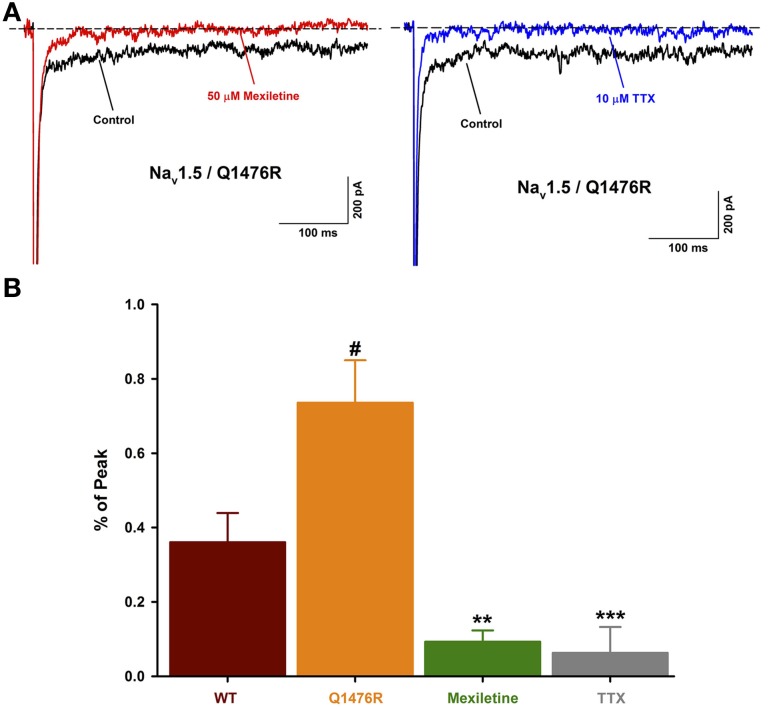
**Effect of Na^+^ channel blockers on the INap. (A)** Representative current traces obtained after a −30 mV depolarizing pulse showing INap before and after drug treatment. The Na_v_1.5/Q1476R traces are depicted in black while the traces after treatment are shown in red for mexiletine (50 μ M, left) and blue for TTX (10 μ M, right). **(B)** Histogram of INap. The INap accounted for 0.36 ± 0.08% (*n* = 13, red column) of the peak current for WT channel and 0.74 ± 0.11% (*n* = 14, orange column) for Q1476R channels (^#^*p* = 0.015 when compared to WT channels). The treatment with 50 μ M of mexiletine or 10 μ M of TTX, respectively reduced the INap to 0.09 ± 0.03% (*n* = 5, green column, ^**^p = 0.002) and 0.06 ± 0.06% (*n* = 5, gray column, ^***^p = 0.001). After treatment, no differences were observed with WT channels. “#” indicate statistical differences when compared to WT channels, “*” indicate statistical differences when compared to Q1476R channels.

The persistent Na^+^ current can be attributed to the Na_v_1.5 channel as it was blocked (0.06 ± 0.06%, *n* = 5) by 10 μ M of tetrodotoxin, a Na^+^ channel blocker (Figure 6). Mexiletine was seen to be efficient to treat the patient. The effect of this class I B antiarrhythmic was thus tested on the persistent Na^+^ current. 50 μ M of mexiletine importantly reduced the persistent Na^+^ current (0.09 ± 0.03%, *n* = 5) (Figure 6).

### *In silico* characterization of the changes induced by the heterozygous mutation Q1476R

The *in silico* characterization incorporates the heterozygous aspect of the mutation. This model is used to observe the effects of the mutation on several parameters including AP, Na^+^ window current INa (the large inward current surge simultaneous with AP upstroke is largely off scale), persistent Na^+^ current (INap), Na^+^ and Ca^2+^ intracellular concentrations (Na_i_ and Ca_i_). As a rule, model simulations were conducted for at least a 2000 s cell lifetime to ensure that SS in all variables was reached.

**Figure 7:**
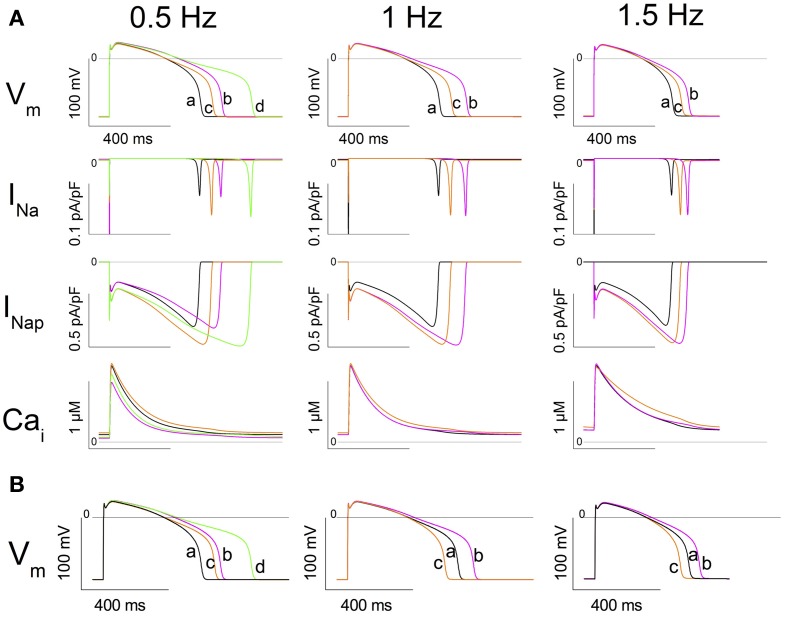
***In silico* simulation of heterozygous cardiomyocytes carrying the Q1476R mutation. (A)** The three columns correspond to different stimulation frequencies indicated at the top of the figure. From top to bottom: the action potential (AP), the fast sodium current (INa), the persistent sodium current (INap), and cytosolic [Ca^2+^] (Ca_i_). Under column 0.5 Hz, traces at SS are superimposed: trace a: WT at 1 Hz (black), trace b: WT at 0.5 Hz (magenta), trace c: WR (1/2 WT + 1/2 Q1476R) at 1 Hz (orange), and trace d: WR at 0.5 Hz (green). Under column 1 Hz, trace a is WT at SS at 1 Hz, trace b is the subsequent AP after changing the model to WR, and trace c is the SS in WR at 1 Hz. Under column 1.5 Hz, trace a is WT at SS at 1.5 Hz, trace b is the subsequent AP after changing the model to WR, and trace c is the SS in WR at 1.5 Hz. **(B)** Model tests of the role of Na_i_ in determining APD_90_. Instead of running the model until SS, Na_i_ is changed instantaneously to its SS value. *Left panel*, trace a shows the SS AP in WT at 1Hz. Trace b shows the subsequent AP recorded after changing Na_i_ to the value previously observed at SS in the WT model at 0.5 Hz (9.84 mmol/L). Trace c shows the subsequent AP recorded from the model after simultaneously setting the model to WR and changing Na_i_ to the value for WR at 1 Hz at SS (12.92 mmol/L). Trace d shows the next AP recorded after changing Na_i_ to the value previously observed at SS in WR at 0.5 Hz (10.95 mmol/L). *Middle panel*, trace a shows the SS AP in WR at 1Hz. Trace b shows the subsequent AP recorded after changing Na_i_ to its value previously observed at SS in the WT model at 1 Hz (11.94 mmol/L). Trace c is the subsequent AP recorded from the model after setting the model to WT. *Right panel*: Trace a shows the SS AP in WR at 1.5 Hz. Trace b shows the subsequent AP recorded after changing Na_i_ to the value previously observed at SS in the WT model at 1.5 Hz (13.59 mmol/L). Trace c shows the subsequent AP recorded from the model after setting the model to WT.

With 1-Hz stimulations, at SS, the WT model shows an APD_90_ of 454 ms and a Na_i_ of 11.94 mmol/L (central column, trace a, in Figure 7A, and Table 2). The model was then changed to the heterozygous condition (denoted as WR), with half of the INa current being WT and half being Q1476R. The first AP obtained in the WR condition had an APD_90_ of 587 ms (trace b) without appreciable change in Na_i_. Both Na^+^ window current and INap amplitudes were increased when the mutation was introduced (2nd and 3rd panels from the top, respectively, central column of Figure 7A). This was consistent with estimates computed from the voltage-clamp data (Figure 5). The difference is that the magnitude of the mutation-induced increase in the window current and in the INap as evaluated in the heterozygous model was two-fold less than that reported in Table 1, since, in the WR model, only half of the current is of the mutated type. When running this heterozygous model to SS (trace c), Na_i_ expectedly increased, reaching 12.92 mmol/L, while APD_90_ shortened to 513 ms. The diastolic Ca_i_ and Ca_i_ transients were also increased (Figure 7A, lower panel, central column and Table 2). The instantaneous APD_90_ lengthening by 29% due to changing from WT to WR condition was reduced to 13% *vs*. WT when the WR model reached SS. Most of the lengthening was thus counterbalanced by changes that occurred during regular stimulations for longer periods (typically 2000 s).

**Table 2:** **APD_90_, Na_i_, and diastolic Ca_i_ at steady state recorded in the model during 1- and 1.5-Hz stimulation**.

**Stimulation frequency**	**Parameter**	**WT**	**WR first AP**	**WR**
1 Hz	APD_90_ (ms)	454	587	513
	Na_i_ (mmol/L)	11.94	11.94	12.92
	Diastolic Ca_i_ (μmol/L)	0.109	0.109	0.135
	APD_90_ in Na_i_ tests (ms)	451	582	
1.5 Hz	APD_90_ (ms)	392	471	436
	Na_i_ (mmol/L)	13.59	13.59	14.57
	Diastolic Ca_i_ (μmol/L)	0.179	0.177	0.221
	APD_90_ in Na_i_ tests (ms)	396	484	

The same simulations were conducted at 1.5 Hz until SS (Figure 7A, 3rd column, and Table 2). APD_90_ and Na_i_, respectively stabilized at 392 ms and 13.59 mmol/L for WT (trace a). After the switch to the WR model, the first AP (trace b) had an APD_90_ of 471 ms (Na_i_ remained unchanged). After reaching SS (trace c), APD_90_ shortened to 436 ms and Na_i_ increased to 14.57 mmol/L. At SS, the diastolic Ca_i_ and Ca_i_ transients had increased in the WR model. The increases were larger than those seen at 1 Hz (Figure 7A, lower panel, 3rd column and Table 2).

To test the effect of bradycardia, the stimulation rate was lowered from 1 Hz to 0.5 Hz. At SS, the WT model had a longer APD_90_ (558 ms) and a lower Na_i_ (9.84 mmol/L) than at 1 Hz. In the WR model at 0.5 Hz, APD_90_ and Na_i_ reached SS values of 705 ms and 10.95 mmol/L (Table 3), while the diastolic Ca_i_ and Ca_i_ transients decreased (Figure 7A, 1st column, lower panel and Table 3). To determine the effect of changes in Na_i_ on APD_90_, changes in Na_i_ were applied instantaneously to the model in several settings (Figure 7B).

**Table 3:** **Effect of bradycardia on APD_90_, Na_i_, and diastolic Ca_i_ at steady state**.

	**WT at 1 Hz**	**WT at 0.5 Hz**	**WT at 1 Hz**	**WT at 0.5 Hz**
APD_90_ (ms)	454	558	513	705
Na_i_ (mmol/L)	11.94	9.84	12.92	10.95
Diastolic Ca_i_ (μmol/L)	0.109	0.057	0.135	0.071
APD_90_ in Na_i_ test (ms)		542	515	683

In the WT model, from SS at 1 Hz, setting Na_i_ to its value at SS at 0.5 Hz produced an APD_90_ of 542 ms (Figure 7B, left panel). This was comparable to the SS APD_90_ at 0.5 Hz when Na_i_ was not imposed (558 ms). Likewise, when SS Na_i_ was imposed in the WR model at 0.5 and 1 Hz, the APD_90_s of the next AP were respectively 683 and 515 ms, vs. 705 and 513 ms at SS when Na_i_ was not imposed (left panel of Figure 7B and Table 3). At 1 Hz, in WR model under SS, changing Na_i_ to WT SS value produced APD_90_ of 582 ms. This value is close to that of the first WR AP (APD_90_ of 587 ms). Then, changing to WT produced APD_90_ of 451 ms which is similar to SS WT APD_90_ of 454 ms (middle panel of Figure 7B and Table 1). At 1.5 Hz (Figure 7B, right panel and Table 2), APD_90_ produced by changing Na_i_ values were very close to those in the original simulations at SS. Thus, applying solely Na_i_ changes reproduced most of the APD_90_ decrease observed at SS.

**Figure 8:**
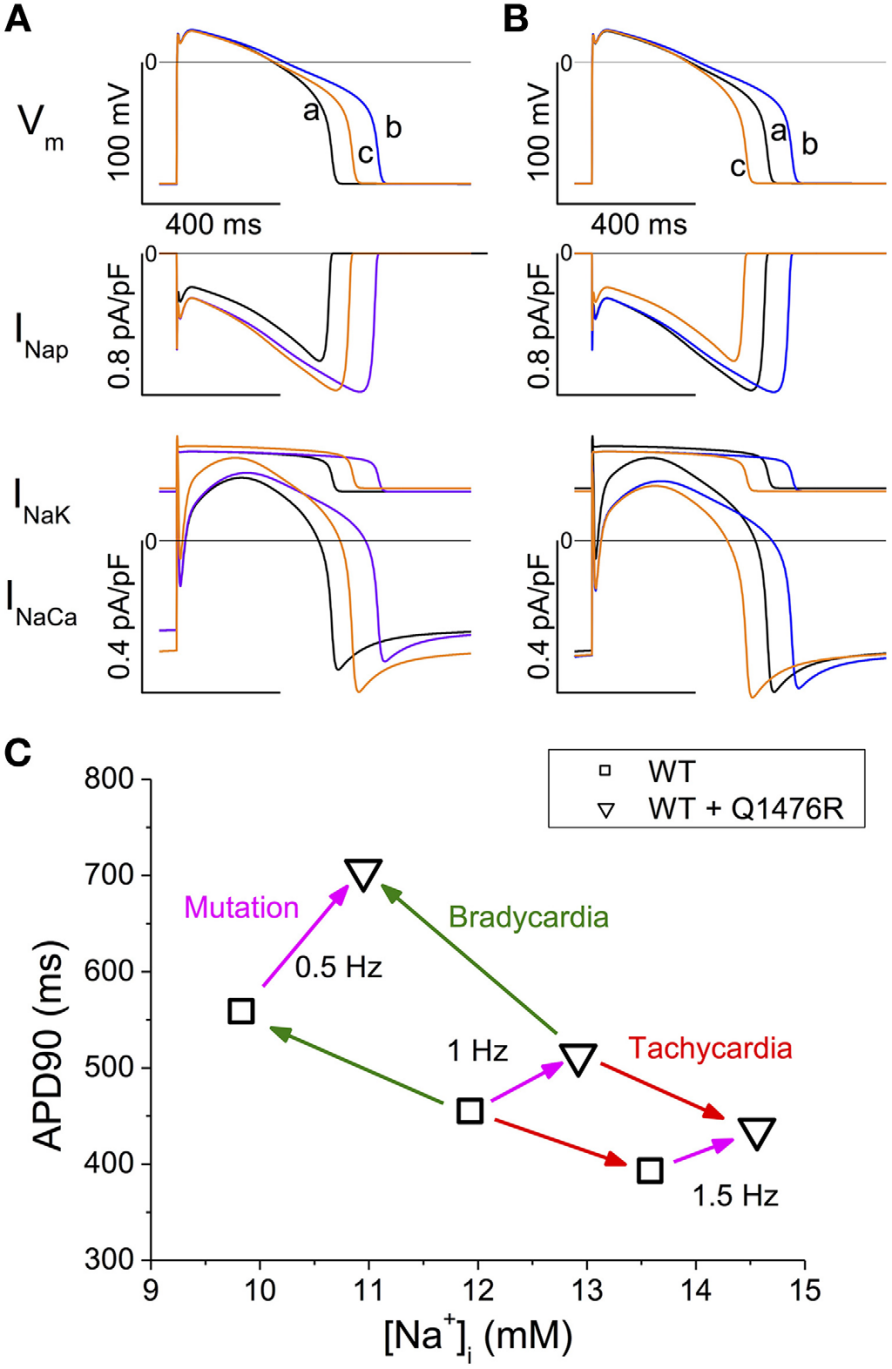
**Time course of INaK and INaCa and summary of SS APD_90_ and Na_i_ values. (A,B)** Analysis of the contribution of INap, INaK, and INaCa to APD_90_ changes. **(A)** The black traces (a) show the SS AP and currents in WT at 1 Hz. The blue traces (b) show the subsequent AP and currents recorded after introducing the heterozygous Q1476R mutation. The orange traces (c) were obtained after reaching the SS. **(B)** The black traces (a) show AP and currents for the WR model stabilized at 1 Hz. The blue traces (b) were obtained when Na_i_ was instantanously set to Na_i_ of the SS WT model. The orange traces (c) were obtained when switching to the WT model. **(C)** Summary of APD_90_ and Na_i_ values at SS at three stimulation frequencies (0.5, 1, and 1.5 Hz) and in the WT and WR models. The green arrows indicate the transitions due to bradycardia, the red arrow those due to tachycardia. The magenta arrows indicate the effect of passing from the WT to WR condition at each frequency.

In the TNNP 2004 model, the only two ion transfer mechanisms that depend on Na_i_ are the Na^+^-K^+^ pump (INaK) and the Na^+^-Ca^2+^ exchanger (INaCa) (Ten Tusscher et al., 2004). Their activity increase when Na_i_ is increased. In Figure 8A, the two upper panels are a repeat of the first and third panels from the top in the middle column of Figure 7. The lower panel of Figure 8A is new and reports the time course of the INaK and INaCa currents during an AP. There was a large immediate increase in the duration of the first AP recorded after switching the model to the WR condition (Figure 8A upper panel, compare traces a and b). This was a direct effect of the increase in the conductance of the INap (middle panel, blue vs. black trace). The magnitudes of the INaK and the INaCa exchanger currents followed the changes in the membrane potential (compare blue and black traces in the lower panel). We further stimulated the model at 1 Hz until SS. The APD_90_ shortened (Figure 8A upper panel, trace c, orange line). While the changes in INap magnitude just followed the changes in membrane voltage values (orange *vs*. blue trace, middle panel), the magnitudes of both the INaK and the INaCa currents increased (orange *vs*. blue trace, lower panel). The increase in the INaK current was small compared to the 30% increase in the magnitude of the INaCa exchanger current.

As seen in Figure 7, there was a concomitant raise in the diastolic Ca_i_ and in the Ca_i_ transients in tandem with the increase in Na_i_ when the stimulations were applied until SS. To isolate the effect of the increase in Na_i_, we started with the WR model stabilized at 1 Hz and instantaneously changed Na_i_ to that of the SS WT model (Figure 8B, black to blue traces). The model was then instantaneously returned to the WT model (Figure 8B, blue to orange traces). The changes in APD_90_, INaK, and INaCa were comparable to those in Figure 8A. This indicates that, when Na_i_ was increased, the reverse mode of INaCa provided the main increase in the outward current that caused the shortening of the APD_90_.

A possible contribution of the increase in window current in the effects of the mutation was tested in the model at steady-state at 1Hz. The diastolic membrane voltage in the WT situation was −85.9 mV and the threshold for stimulation was 21.4 pA/pF, whereas in the WR situation, they were −85.5 mV and 20.8 pA/pF, respectively. Thus, there was a 0.4 mV depolarization and a 2.8% decrease in stimulation threshold. Further, when the window current of the WR model was made identical to that in the WT model, by suppressing any changes in steady-state activation and inactivation curves, the diastolic membrane voltage was −85.7 mV and stimulation threshold 21.3 pA/pF. Thus, although slightly, the window current appeared to increase cell excitability.

## Discussion

We biophysically characterized a novel LQT3 mutation (Q1476R) found in a previously symptomatic 34-year-old female with a classic LQT3 ECG phenotype. The Q1476 residue is a highly conserved amino acid in all Na^+^ channels (Figure 2) throughout evolution, from humans to zebra fish. Interestingly, a second highly conserved Q residue (Q1475) (Figure 2) was found immediately upstream from Q1476. These two residues were located in the inactivation gate upstream from the IFM motif. Given their location, they are likely to play a role in the inactivation process. Moreover, the Q1489H missense substitution on *SCN1A* encoding the Na_v_1.1 neuronal voltage-gated Na^+^ channel (the Q1475 homolog in Na_v_1.5) is highly expressed in the central nervous system, including the retina, and is found in patients with familial hemiplegic migraine (FHM, Figure 2B) (Vahedi et al., 2009). Unfortunately, no biophysical characterization has been reported for this mutation. We can speculate that a gain-of-function due to this mutation may be the cause of FHM.

The present paper provides support for the concept that the increase in the window current and the increase in the TTX sensitive persistent current due to the LQT3 mutation (Q1476R) cause LQTS. This is consistent with all studies indicating that mutations in *SCN5A* causing LQTS are related to a gain-of-function (Schwartz et al., 2012).

The treatment with 50 μ M of mexiletine importantly reduced the INap observed on Q1476R mutant channels. This might explain the beneficial effects of mexiletine treatment on the patient. Unfortunately, the nausea due to mexiletine have imposed the discontinuation of the treatment.

As expected from its localization, the mutation impacts the inactivation process. The Q1476R mutation resulted in a depolarizing shift of inactivation, which tended to increase the overlap of activation and inactivation gating (Figure 5). At voltages within this overlap region, Na^+^ channels are partially but not fully inactivated, thus increasing the potential for persistent window currents (Attwell et al., 1979). At −50 mV, the peak window current probability predicts that a small percentage (0.1%) of the mutant channels will be persistently activated. The resulting inward Na^+^ current at resting membrane potentials could depolarize cardiac myocytes, leading to an increase in their excitability. The increase in persistent current is not surprising as the mutation is localized in the inactivation gate (DIII-DIV linker). The effect of this mutation on the inactivation process would be a decrease of the probability for channels to enter an inactivated state. Consequently, a small amount of Na_v_1.5 Q1476R channels will not inactivate after the rapid activation. Similar mechanisms are believed to underlie the increased excitability of sensory neurons harboring inherited human pain disorder mutations that produce shifts in Na_v_1.7 activation and inactivation of similar polarity and magnitude as those observed in the present study (Harty et al., 2006; Rush et al., 2006; Dib-Hajj et al., 2010).

The *in silico* model confirmed the arrhythmogenic potential of the Q1476R mutation. When the model was changed to the heterozygous mutated paradigm, there was an immediate effect that prolonged APD_90_. Continued stimulation caused a partial reversion of this effect when SS was reached. At the same time, both Na_i_ and Ca_i_ increased. Figure 8C summarizes the effects of the mutation combined with the heart rate on APD_90_ and Na_i_. At SS, the accumulation of Na^+^ ions, related both to an elevated frequency and to the presence of the mutation, tended to shorten APD_90_. This would in turn tend to moderate the LQT effect of the mutation at elevated heart rates such as under effort or moderate stress. The gain-of-function due to a persistent Na^+^ current may thus protect against arrhythmogenesis related to a limited increase in heart rate. However, as was seen in the model, an overload of Ca_i_ stores may appear at higher rates and may trigger arrhythmias (Christe et al., 2008). Our data are consistent with published experimental and model studies (Pieske and Houser, 2003; Noble and Noble, 2006; Undrovinas et al., 2006; Soliman et al., 2012). When combined with elevated Ca^2+^ entry under adrenergic stress, arrhythmias may appear due to early or late after-depolarizations. Under elevated Ca_i_ conditions, a decrease in the L-type Ca^2+^ current ought to take place due to the decrease in the transmembrane [Ca^2+^] gradient and to the accelerated decay by calcium-dependent inactivation. Nevertheless, its contribution to the overall effect should be minor. The slowly activating potassium current IKs was shown to be increased by elevation of Ca_i_, as due to a relief of KCNQ1 inactivation by calmodulin in a Ca^2+^ dependent way (Ghosh et al., 2006). This feature was absent in the model. Such an effect would enhance the frequency-dependent effect observed in the model, resulting into a further shortening of the AP at high frequencies that would be larger in the WR than in the WT situation.

**Figure 9:**
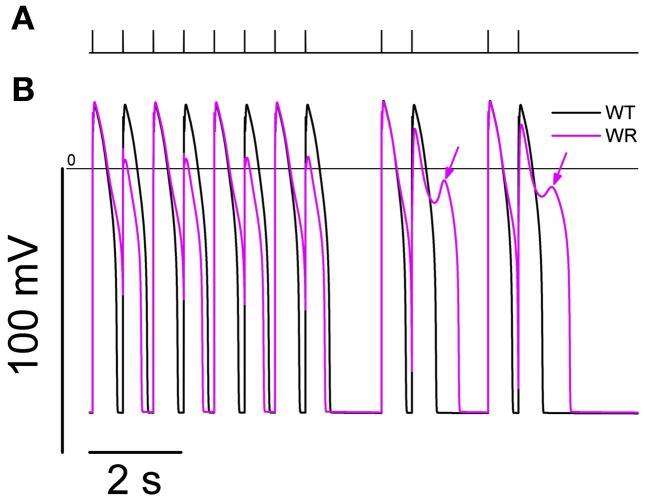
**Simulation of an interval variations paradigm known to trigger EADs and *Torsades de Pointes*.** From SS at 0.5 Hz, a series of 40 stimulations were applied at 666 ms intervals. Then, a combination of a 1666 ms interval plus a couple of stimulations at 666 ms interval was applied twice (panel **A**). The action potentials produced by model for the last 12 s of the protocol are shown in **(B)**. While the WT model (black line) shows a separate AP in response to each stimulation, the WR model APD_90_ (magenta line) was longer than the 666 ms interval, causing a bigeminal aspect: every second AP is smaller and shorter. After the pauses, EADs were present in the WR model only (arrows).

A combination of interval variations known to trigger EADs and *torsades de pointes* (El-Sherif et al., 1999; Viswanathan and Rudy, 1999; Tan et al., 2006) has been tested in the model. EADs appeared in the WR condition while the WT remained unaffected (Figure 9). This is consistent with emotional stress and exercise as known triggers of arrhythmias in LQT3 (Schwartz et al., 2001).

Conversely, bradycardia causes a lower Na_i_ and a lengthening of APD, which are well-known features of bradycardia (Faber and Rudy, 2000). Changes in diastolic Ca_i_ and Ca_i_ transients are also observed. Each of these may change the magnitude of ionic currents and exchangers. Elevated Na_i_ is known to cause an activation of the Na/K ATPase pump and an increase in the reverse mode Na-Ca exchanger current. Both of these processes may shorten APD (Armoundas et al., 2003), as well as the increase in Ca^2+-dependent^ inactivation of the Ca^2+^ current due to elevated Ca_i_ (Carmeliet, 2006). Our simulations showed that most of the APD changes were due to changes in Na_i_, mainly through a lower INaCa. The LQT effect of the mutation is likely to be more serious at low heart rates, as illustrated in our simulations where APD_90_ reached very critical values around 700 ms in the heterozygous model. Thus, the carrier of the mutation may be preferentially sensitive to factors triggering arrhythmias when at rest, which is a characteristic of LQT3 syndrome. During activity, APD shortening, due to increased entry of Na^+^ ions, may protect the carrier against arrhythmias. These results should be qualitatively reproduced in other models of human ventricular myocytes, since the basic features of the Na_i_ homeostasis ought to obey to the same constraints. Furthermore, the functional consequences, as analyzed here for mutation Q1476R, may in principle be extended for other LQT3 mutations involving an increase in persistent and window currents. The intracellular Na^+^ overload, as related to the gain-of-function in Na^+^ channels, is proper to LQT3 and is unlikely in LQT syndromes related to potassium channel dysfunctions. This might be taken into account for personalized prevention and therapy of LQT3 patients.

### Limitations of the *in silico* model

The basic TNNP model assumed no fraction of INap. However, the magnitudes of the INaCa, INaK, and Na^+^ background currents had been adjusted to account for the high Na_i_ in ventricular human myocytes. The present modified model may thus have an overall excess of Na^+^ entry. A late Na^+^ current was added to the model in the WT condition, and its magnitude was limited to one-third of the fraction of the peak Na^+^ current found in tsA201 cells. This caused the APD_90_ in the WT configuration to be 454 ms vs. 325 ms in the basic TNNP model, whereas values of ~370 or 400 ms have been reported by Li et al. (1998, 1999) in human ventricular myocytes. As a result, the effects of the mutation may have been overemphasized in the present simulations.

Several ion channels have been shown to be modulated by intracellular Ca^2+^, either directly or through intracellular effectors (e.g., calmodulin), such modulations were not included in the model. Several additional channels (e.g., stretch-sensitive channels) found in mammalian ventricular myocytes were neither included. A number of investigations in human ventricular myocytes are still needed to document their formulation.

Lastly, this model assumes that no changes other than those due to the Na^+^ channel mutation took place. There are reasons to doubt this, since a permanent Na^+^ and Ca^2+^ overload, as seen here in the WR model, are known triggers of remodeling of ion channel densities and excitation-contraction coupling efficiency in cardiac cells, which may have taken place over the long-term in carriers of the mutation.

### Conflict of interest statement

The authors declare that the research was conducted in the absence of any commercial or financial relationships that could be construed as a potential conflict of interest.

## Appendix

Equations used in the TNNP model:

WT is a variable taking three possible values: WT = 1 for a pure WT cell, WT = 0.5 for the heterozygous condition and WT = 0 when all channels are mutated.

$$g_{\text{NaWT}}=\text{WT}\cdot 14.838$$

$$g_{\text{NaQ1476R}}=\left(1-\text{WT}\right)\cdot 14.838\cdot \left(1-0.02\right)$$

$$i_{{\text{Na}}_{\text{fastWT}}}=g_{\text{NaWT}}\cdot m_{\text{WT}}{}^{3}\cdot h_{\text{WT}}\cdot \left(V-E_{\text{Na}}\right)$$

$$m_{\text{infWT}}=\frac{1}{1+e^{\frac{V+53.6}{-7.4}}}$$

$$i_{{\text{Na}}_{\text{fastQ1476R}}}=g_{\text{NaQ1476R}}\cdot m_{\text{Q1476R}}{}^{3}\cdot h_{\text{Q1476R}}\cdot \left(V-E_{\text{Na}}\right)$$

$$m_{\text{infQ1476R}}=\frac{1}{1+e^{\frac{V+51.3}{-8.2}}}$$

$$\tau_{m}=\frac{0.5\cdot \left(\frac{1}{101.6\cdot e^{V\cdot 0.1135}+0.022,68\cdot e^{-0.0717\cdot V}}+0.0001\right)}{4.83}$$

$$h_{\text{infWT}}=\frac{1}{1+e^{\frac{V+86.5}{4.4}}}$$

$$h_{\text{infQ1476R}}\frac{1}{1+e^{\frac{V+80.6}{4.3}}}$$

$$\tau_{h}=\frac{0.5\cdot \left(\frac{1}{1.1381\cdot 10^{-6}\cdot e^{-0.1017\cdot V}+6.537\cdot e^{0.08016\cdot V}}+0.0005\right)}{4.83}$$

$$g_{\text{NapsWT}}=\frac{\text{WT}\cdot 14.838\cdot 0.0019}{fm}$$

fm = 3

$$g_{\text{NapsQ1476R}}=\frac{\left(1-\text{WT}\right)\cdot 14.838\cdot \left(1-0.02\right)\cdot 0.0033}{fm}$$

$$i_{\text{NapsWT}}=g_{\text{NapsWT}}\cdot m_{\text{infWT}}{}^{3}\cdot \left(V-E_{\text{Na}}\right)$$

$$i_{\text{NapsQ1476R}}=g_{\text{NapsQ1476R}}\cdot m_{\text{infQ1476R}}{}^{3}\cdot \left(V-E_{\text{Na}}\right)$$

$$i_{\text{Na}}=i_{\text{NafastWT}}+i_{\text{NafastQ1476R}}+i_{\text{NapersWT}+NapersQ1476R}$$
